# A qualitative study on barriers and enablers to uptake of diabetic retinopathy screening by people with diabetes in the Western Province of Sri Lanka

**DOI:** 10.1186/s41182-019-0160-y

**Published:** 2019-05-17

**Authors:** Mapa Mudiyanselage Prabhath Nishantha Piyasena, Gudlavalleti Venkata S. Murthy, Jennifer L. Y. Yip, Clare Gilbert, Tunde Peto, Mahesh Premarathna, Maria Zuurmond

**Affiliations:** 1 Clinical Research Department, Department of Infectious and Tropical Diseases, International Centre for Eye Health, London School of Hygiene and Tropical Medicine, London, WC1E 7HT UK; 2 School of Medicine, Dentistry and Biomedical Sciences, Queen’s University, 97 - Lisburn Rd, Belfast, BT9 7BL Northern Ireland; 3 Department of Sociology, University of Colombo, Reid Avenue, Colombo 03, Colombo, Sri Lanka; 4 Clinical Research Department, Department of Infectious and Tropical Diseases, International Centre for Evidence in Disability, London School of Hygiene and Tropical Medicine, London, WC1E 7HT UK

**Keywords:** Barriers, Diabetes mellitus, Diabetic retinopathy, Screening, Sri Lanka

## Abstract

**Background:**

Blindness and visual impairment from diabetic retinopathy (DR) are avoidable through early detection and timely treatment. The Western Province of Sri Lanka has the highest prevalence of diabetes mellitus (DM) (18.6%) in the country. A situational analysis identified a significant gap in DR screening services (DRSS) uptake in this region. Barriers that hinder people with DM (PwDM) from attending DRSS are poorly understood. The purpose of this study is to understand the factors which influence the uptake of DRSS and follow-up to inform health promotion strategies and improve the uptake of these services.

**Methods:**

Eleven focus group discussions (FGDs) were conducted with PwDM who presented to medical, general eye and vitreoretinal services in three public sector institutions (two tertiary and one secondary level) in the Western Province between October 2016 and March 2017. We enrolled six groups (four Sinhala speaking, two Tamil) of women and five groups (three Sinhala and two Tamil) of men representing ethnicity and gender. We performed a thematic analysis and described the main themes and subthemes using the socio-ecological model as a framework.

**Results:**

We identified lack of knowledge of both the condition and the need for screening as key barriers to access DRSS. Socio-cultural factors in the family environment, economic reasons and institutional factors were also important barriers. Additional reasons include long waiting time at eye clinics and poor referrals exacerbated by the lack of a systematic DRSS. In addition, attitudes to DRSS such as fear of discomfort from the procedure and the need for accompaniment following mydriasis were also deterrents to follow-up screening.

**Conclusion:**

This study has shown that there are inter-related user, family, and institutional factors which affect the uptake of DRSS. Understanding how DR is conceptualised by PwDM in this region is essential to refine strategies to improve access to DRSS. Strategies to improve knowledge need to be more culturally acceptable and relevant to PwDM and their families, with increased availability of DRSS at convenient locations may increase timely uptake of screening.

**Electronic supplementary material:**

The online version of this article (10.1186/s41182-019-0160-y) contains supplementary material, which is available to authorized users.

## Background

Diabetes mellitus (DM) is an emerging global epidemic. The International Diabetes Federation estimated that there will be 629 million people with diabetes (PwDM) by the year 2045 [1]. Diabetic retinopathy (DR) is a common microvascular complication of DM potentially leading to visual impairment and blindness. DR has an asymptomatic stage that can go unnoticed until it affects vision leading to blindness [2]. Several studies have shown that good control of blood glucose levels and hypertension and DR screening, with timely identification and treatment of significant retinal changes, reduce the progression of sight-threatening DR [3–7]. However, delivering an effective screening programme with a high level of coverage is difficult even in high-income settings [8].

Sri Lanka is a lower middle-income country which has a distinctive and sustainable health system. Sri Lanka has achieved a remarkable development in health indicators compatible with the millennium development goals and a high literacy rate (> 10 years of age, males 96.9%, females 94.6%) compared to neighbouring countries in the region [9, 10]. The country has a population of 20.2 million (2012), 5.82 million (28.7%) of whom live in the Western Province [10]. This province has three districts namely, Colombo, Gampaha and Kalutara, with several different ethnic groups. Colombo is the most densely populated city in Sri Lanka with 3428 persons/km^2^ [10]. Health care in Sri Lanka is provided at the point of delivery in the public sector, without needing a referral from a general practitioner and eye care is free. Individuals of middle or high socio-economic status tend to favour the private sector, including for DM management and eye care.

The crude prevalence of DM in Sri Lanka was estimated at 12.6% (age > 20 years) as reported in a national level survey with the highest prevalence (18.6%, 95% CI 15.8–21.5%, age > 20 years) in the Western Province [11]. The prevalence of any DR among PwDM ranged from 18.1% (mean age 37.1 years) to 27.4% (mean age 56.4 years) [12, 13]. A situational analysis of the Western Province in 2014 indicated a wide gap between the background need and screening provision for DR, with an estimated additional 670,970 DR screening visits and 110,690 laser procedures which need to be performed to prevent sight loss due to DR per year to address the unmet need [14]. Sri Lanka does not have a systematic screening programme for DR, but PwDM who attend out-patient medical care are given a referral letter for an annual retinal examination at the nearest eye clinic [14]. Clinicians in the Western Province report significant numbers presenting with more severe stages of DR, leading to costly eye surgeries and poorer outcomes. This is a burden to the health system, leading to long waiting time for surgeries, extending beyond 1–2 years.

Access to health care depends on a complex interaction of various factors. The availability of screening services will inevitably influence uptake [15]. Studies that explored eye health-seeking behaviour and barriers to access of DR screening services (DRSS) by PwDM have identified a range of socio-cultural factors which are likely to be context specific. Barriers including low economic status [16, 17], low level of literacy [18] and other socio-economic inequities in access [19] affect the uptake of eye care services [20, 21]. Low levels of awareness and knowledge among the PwDM about DR and its screening is another common barrier [22–25]. However, there are no known studies which have looked at the specific barriers in the Sri Lankan context, and this study addresses this gap.

## Methods

### Aim

The aim of this study was to explore why PwDM do not take up referral for free eye examinations in the Western Province of Sri Lanka, from the patients’ perspectives. We were interested in identifying the barriers in the care pathway in this local context. This study was conducted as part of a larger feasibility study, to develop an integrated DRSS programme in Sri Lanka. We assumed that identifying barriers for PwDM will enable us to make recommendations for a systematic DRSS strategy in Sri Lanka and to inform the development of health education interventions to facilitate access.

### Conceptual framework

**Fig. 1:**
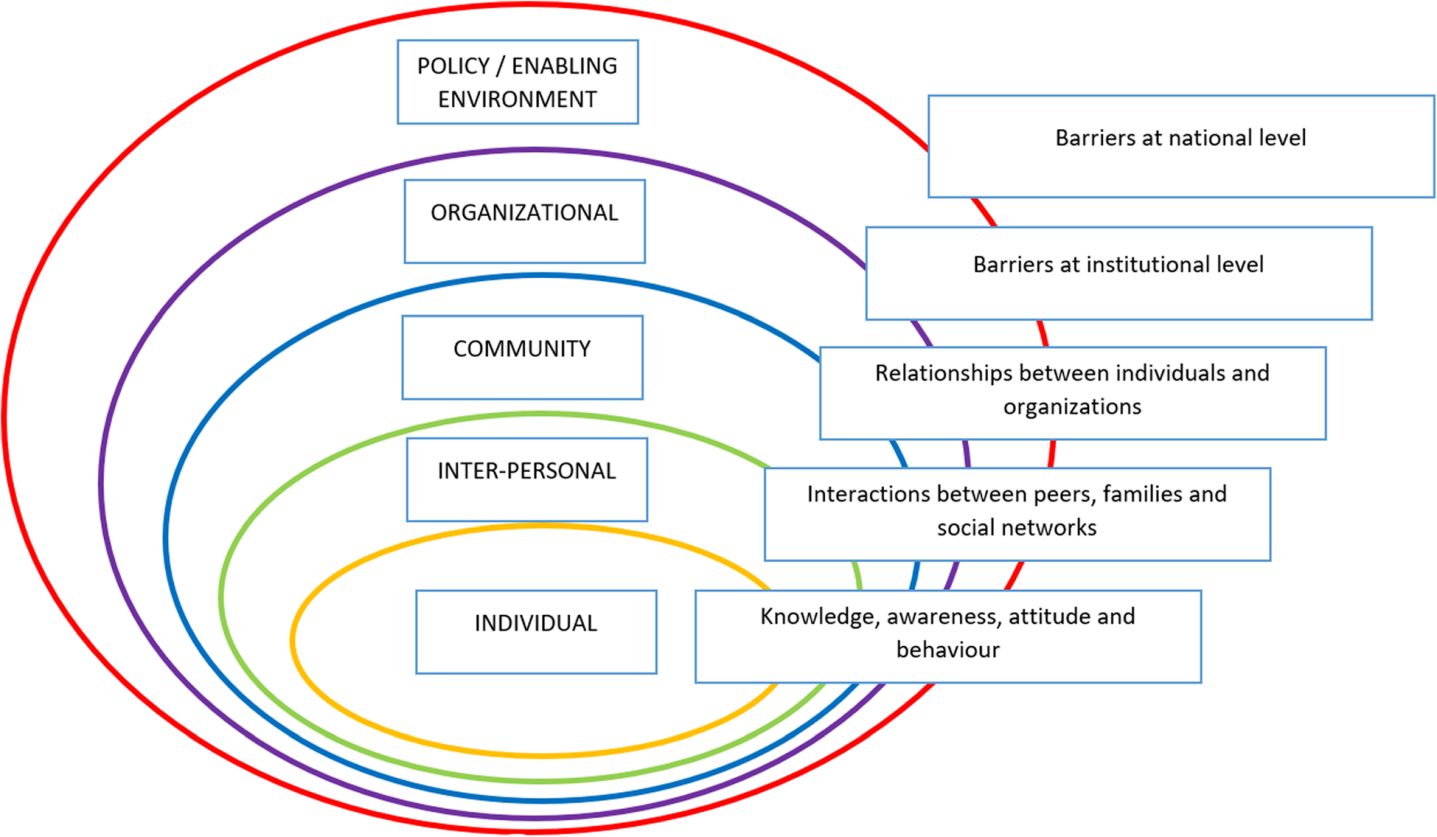
Illustration of socio-ecological model to understand interactions of PwDM and environment depicting barriers at each level

We used the “Socio-Ecological Model” framework to analyse the study. This model describes dynamic interactions among and between various personal and environmental factors and their impact on an intended outcome [26, 27]. We used this model to develop our understanding of the multi-faceted interactions between individuals (PwDM) and their environment and therefore explain patients’ behaviour in relation to access of DRSS. This model was also used for examining barriers within the different layers of the individual, family and society, including interactions with the service providers (see Fig. 1) [28, 29].

### Research team and reflexivity

The team of investigators comprised the lead investigator (MMPNP), four moderators (three males and one female) and two research assistants (one male and one female). The moderators were all experienced Sri Lankan sociologists, and each of them was fluent in either Sinhala or Tamil language. The research team spent a few hours at a study centre observing the clinics before conducting focus group discussions (FGDs). The objective of non-participatory observations was to identify the processes involved in managing a PwDM and for the sociologists to familiarise themselves with the context. The FGDs were conducted in a closed room of the hospital to maintain privacy. Each FGD lasted between 45 and 90 min (the topic guide available as Additional file 1).

The topic guide for FGDs was informed by a literature review, translated into the two key languages, pilot tested and then revised. It explored knowledge, awareness and socio-economic and cultural factors that could affect the DRSS health seeking behaviour of a PwDM.

### Study sites

We purposefully selected urban public sector clinic settings in two districts of the Western Province: two tertiary care institutions (one multi-speciality and one eye hospital in Colombo district) and one secondary level institution (a general hospital in Gampaha district). These clinics are all attended by a large number of people every day, most commonly those with chronic disease and lower socio-economic position and are in urban settings [30].

### Participant selection

Potential participants at the out-patient clinics were asked to complete a short questionnaire by study research assistants, whilst waiting for their consultation. We used the completed questionnaires to purposively sample participants > 18 years of age to ensure representation from different ethnic groups (Sinhala, Tamil and Moor), men and women, different economic and educational backgrounds and at different stages of care or different stages of diabetic eye disease, ranging from no DR to those already receiving treatment for DR. DM and DR status were determined by referring to the medical records, and socio-economic position was assessed using the household income categories of the population census. Eleven FGDs were held with a total of 87 participants. These FGD were conducted separately according to the gender and ethnicity and language. The Moor minority ethnic group speak the Tamil language and were combined to these FGD due to pragmatic reasons. Seven FGDs in Sinhala and four in Tamil were conducted (see Table 1).

**Table 1:** Composition of focus groups

Medium of discussion-Sinhala language		Medium of discussion-Tamil language	
Female	Male	Female	Male
Group 1: in medical care *N* = 9	Group 5: in medical care *N* = 7	Group 8: in medical care *N* = 5	Group 10: in medical care *N* = 9
Group 2: in medical care *N* = 9	Group 6: had been referred to an eye clinic *N* = 10	Group 9: mixed group: had been referred to an eye clinic or who had previous DR treatment and major surgery *N* = 5	Group 11: mixed group: had been referred to an eye clinic or who had previous DR treatment and major surgery *N* = 6
Group 3: Had been referred to an eye clinic *N* = 6			
Group 4: had previous DR treatment and major surgery *N* = 12	Group 7: had previous DR treatment and major surgery *N* = 9		

### Analysis

A thematic analysis was conducted in the two main local languages. Audio records were transcribed into local languages, and two separate researchers coded (in Sinhala and Tamil) data after familiarising themselves with the content. Afterwards, the coding was cross-checked by a sociologist (MP) and experienced qualitative researcher. All data under a theme were further analysed in detail and categorised into subthemes and tabulated. Further triangulation of data was conducted by a second reviewer (MMPNP). The main themes, sub-themes and relevant quotations that emerged were translated into English for this paper.

## Results

### Description of the sample

Eighty-three percent of the participants were > 50 years of age (mean 58.7 years ± 1.12), all had type 2 DM (mean duration of DM 9.5 years ± 0.75, mean age at DM diagnosis 48.8 years ± 1.39), and 68% were from lower socio-economic background, as identified through the house-hold income level. Ninety-two percent had education up to primary and above. Fifty-two percent were women, 72.4% were Sinhalese which reflects the proportion in Western Province and a mix of those from urban (48.3%) and rural areas (51.7%). On average, participants lived between 10 and 20 km away from the hospital. Twenty-four percent of the participants had not had any previous examination. Approximately one-fifth (18.3%) presented late and were found to have more severe late stage of DR (tractional retinal detachments) and had previously received major eye surgeries (see Table 2).

**Table 2:** Participants’ characteristics

Variables	Data
Gender	Male *n* = 42 (48.3%)
	Female *n* = 45 (51.7%)
Age (years)	Mean 58.7 years
	Range (26–79) years
Duration of diabetes (years)	Mean 9.6 years
	Range (1–28) years
Age at diagnosis of diabetes	Mean 48.9 years
	Range (20–70) years
Ethnic group	Sinhalese *n* = 63 (72.4%)
	Tamil *n* = 18 (20.7%)
	Moors *n* = 5 (5.8%)
	Other *n* = 1 (1.1%)
Main language	Sinhala *n* = 61 (70.11%)
	Tamil *n* = 26 (29.89%)
Level of education	No school *n* = 7 (8.1%)
	Primary *n* = 26 (30.2%)
	Secondary *n* = 12 (13.9%)
	GCE *n* = 39 (45.3%)
	Degree and above *n* = 2 (2.3%)
Income (per month)	LKR < 39,220 *n* = 31 (35.6%)
	LKR (39,220–69,880) *n* = 28 (32.2%)
	LKR > 69,880 *n* = 28 (32.2%)

### Knowledge and awareness

One of the main barriers to accessing DRSS was a lack of awareness and knowledge about DR among PwDM. This included low levels of knowledge that DM could lead to loss of vision including blindness and a lack of understanding among those who has vision loss that visual impairment was attributable to DM. Although most participants had a vague idea that DM could affect the eyes, their knowledge of DR blindness was basic.

I do not know how diabetes causes loss of vision. I do not know how to tell more about it (FGD 1, female (F), Sinhala speaking(S)).

Most PwDM understood that DM was a disorder of the blood, and they generally called diabetes “sugar” in the local language. However, there was limited understanding of the causal link between “blood sugar” levels and how this could lead to vision problems.

It was more common in the Sinhala FGDs for the reduced vision to be explained by a weakness in the small blood vessels, “nahara” (tubes) in the local language. The Sinhalese often correlated diseases of any organ as weakness in the blood vessels. In contrast, it was more common in the Tamil FGDs for the loss of vision to be attributed to God as illustrated in the following quotation:

God will decide what will be given to us, If God has thought that it is better not to give diseases to this person … .that is His decision. If god has given an illness to you, you cannot refuse it. You will have to ask from the god to take it back … So we have to pray to the God to heal the disease. (FGD 8, F, Tamil speaking (T)).

Vision problems were frequently explained as being caused by cataracts, the need for glasses or glaucoma, all of which are “conditions” familiar to the local population. Therefore, many PwDM thought that undergoing cataract surgery and wearing spectacles would solve their eye problems. We detected considerable confusion around the different types of eye conditions; some Sinhala participants mistakenly conflated DR with glaucoma, mentioning the word “glucose”. We also found poor understanding of different structures within the eye, such as the retina, which are not visible, and which further impeded their understanding of the disease.

I got to know that when diabetes increased you get glaucoma. I think glaucoma means increased glucose in your blood. Because of that you become blind. (FGD 5, Male (M)-S).

In contrast, FGDs conducted with PwDM in the vitreoretinal clinics, who had already experienced sight loss and treatment, had better comprehension of the condition. They generally indicated that their awareness grew after experiencing symptoms and treatment, as illustrated by a 54-year-old man who recently received surgery.

After you lose sight, it is very difficult to restore, whatever you do. The reduced vision will remain for ever. Even if you put a lens (intra ocular lens implantation) you cannot take back your previous good vision. I underwent a big surgery recently, as there was blood inside my eye ball. Now I know it is difficult to cure. (FGD 7, M-S).

Poor understanding of asymptomatic early stages of DR was a related sub-theme. Participants described suffering from other illnesses, lack of visual symptoms or discomfort in their eyes affected their DRSS uptake, illustrated in one of the female FGDs: “I do not want to rush to check my eyes since I do not feel any problems in my eyes”*.* Participants were reluctant to take actions when there was no immediate threat to life, and health-seeking behaviour was influenced by personal experiences of visual symptoms in the past, such as reduced vision or vision loss. They were not aware of treatment options available to manage DR.

“When you say chest pain, you are scared …. When you say kidney problem, you are scared. When you say you would get reduced sight, you would try to correct it with glasses and any how try to see. If you can see with the glasses, you would not have much concern about it”. (FGD 2, F-S).

### Socio-cultural and economic factors

The socio-cultural environment also impacted decision-making to access services. The subthemes included responsibilities of looking after family members, domestic work and the patriarchal role of other male family members in determining women’s access to eye clinic/the hospital. There were considerable gender differences, reflecting societal and gender norms in Sri Lanka.

Data collected in female FGDs revealed societal values as barriers to attending DRSS. There was evidence that the traditional patriarchy dictated decisions on activities and spend by family members. Women were further subordinated by their own perceptions as they commonly stated that they did not like to be a burden on other family members, even for health matters, because their role was to serve the family. Further, they were commonly not in a position to prioritise their own health care, when there were many responsibilities at their home environment, a theme that did not emerge in the male FGDs.

Though I have an appointment date [to check eyes], I was not able to go due to some reason, mainly problems at home. … suddenly children get ill … .or children say there is a parents’ meeting at school. (FGD 2, F-S).

Because of problems and day to day work load at home, I couldn’t go [to the eye clinic]. When we are ploughing the paddy field, I have to prepare meals for the workers, also I have to accompany my son to the school. Because of this and that reason I could not go. (FGD 9, F-T).

In contrast, it was more common for the male FGDs to offer economic reasons as a barrier, citing financial constraints. They saw their family role as a breadwinner. Under these circumstances, financial constraints, difficulties in obtaining leave from work and loss of daily earning were the main barriers to attending DRSS. The fact that they had to attend the clinic at least two or three times to complete a full eye examination, often with long waiting queues, further exacerbated the loss of earnings. In this work priority environment, men prioritised income generation over accessing DRSS especially given the asymptomatic nature of early DR.

I have a small tea kiosk in Pettah (Colombo) … I cannot close it even for a single day. It is a very small income. However, I would lose that amount also if I close the stall. Therefore, I do not have much time to attend a clinic. (FGD 11, M-T).

When my father died, I was eleven. Since the age of 11, I worked and looked after my family members … So, I have to earn my expenses to look after them …. Therefore, I could not care much about my health. I am a mason and I work 24x7 continuously. I did not have time to go to check my eyes. (FGD 7, M-S).

### Institutional factors

Patient experience in clinics and hospitals also shaped people’s willingness to take up referrals. One sub-theme was the poor organisation of care, including very long waiting times, some even waiting a whole day without eating, crowded and uncomfortable waiting areas with limited seating and confusing appointment systems which impeded efforts to rebook a missed appointment.

I went to check my eyes at X hospital. I came back without checking my eyes after seeing the large crowd there. (FGD 8, F-T).

There are long queues. So, it is very difficult to find a place to sit as there are many people. Also, there are no chairs to sit. There is no proper canteen to have a meal. We have to bring our own water bottle. (FGD 3, F-S).

Other sub-themes related to their experience with the doctors at medical/eye clinics. Participants reported very limited time for consultation, poor referrals and limited counselling for how to follow up their screening test. PwDM showed poor appreciation of the value of regular screening, especially when the screening outcome was negative.

I was asked to go to Y hospital and checked my eyes. So, I went there once, and they checked everything and told me that nothing was wrong with my eyes. Afterwards they gave me a letter to come back, But I did not go back, I thought, there was no need to check again, since they told me that my eyes were alright (FGD 7, M-S).

Poor experience of previous eye examination such as discomfort of the dilating eye drops and reduced vision after dilating, in particular, the resulting need for a companion to the clinic, appeared to be another hurdle which they had to negotiate within the family that may prevent them from attending again.

It is very difficult after putting the drops and very difficult to see when you go back home under bright sunlight … it is really blurring … ..I usually do not go for checking if there is no one to accompany. You cannot do this and come alone afterwards. (FGD 3, F-S).

Overall, participants described various inter-related factors which contributed to their decision to decline or to delay attending screening services. We found evidence of an interplay of societal, institutional and personal and inter-personal factors that contribute to poor attendance of DRSS.

## Discussion

This study explored barriers to access of DRSS by PwDM in the Western Province of Sri Lanka, which revealed barriers at the individual, family and institutional levels. We found that lack of knowledge and awareness, socio-cultural, economic and institutional factors were the main domains of detected barriers. Individual-level barriers identified include poor understanding of DR characteristics which resulted in low uptake of screening as well as poor follow-up. Other studies have also shown that lack of knowledge and awareness about DR forms a barrier to uptake of DRSS in low- and middle-income countries [22, 23] and high-income countries [31–33]. The “St Vincent declaration” states that plans for the prevention, identification and treatment of DM and its complications should be implemented as it is a growing problem [34]. However, these targets were not achieved in most of the low- and middle-income countries.

We observed that the absence of colloquial words for “diabetic retinopathy” and “retina” in local languages and common use without understanding of bio-medical jargon contributed to patients’ misunderstanding, further aggravated by the short consultation time in clinics and the use of English language terms by the doctors without taking time to explain. Providers were reported to have used the English term of “diabetic retinopathy” when describing the condition. Some PwDM confused “diabetic retinopathy” with “glaucoma”, possibly due to the homophonic syllables in “glucose” and “glaucoma”. The confusion between the terms could also be attributed to health promotion activities on glaucoma in this region. The perceived disconnect between DM, sugar levels and the effects on the eye may be a key target to improving the knowledge of the PwDM on DR.

The misconceptions on how and why a screening programme is delivered and deterred access have been observed in other studies. One UK study found some PwDM confused DRS with retinal photographs taken during routine eye examinations at optometrists [31]. The reason for annual eye examinations was also reported to be poorly understood in other studies [35, 36]. The particular challenge of understanding the importance of regular checkups in the asymptomatic stage is also not new and has been shown in several other studies in low-income [24, 37–39] as well as in high-income countries [40–43]. The early asymptomatic phase has similarly been observed as a barrier to access services in the eye condition of glaucoma [44, 45]. It is a challenge for the providers to convince an apparently healthy person to participate in routine screening programmes in the absence of a perceived threat to sight. The asymptomatic nature of DR was shown to be an important element in health promotional material [46]. An individual’s better understanding of their susceptibility to vision loss may increase motivation to attend a screening examination.

Our study showed that people with advanced proliferative DR, such as tractional retinal detachment, and who had undergone treatment had, perhaps not surprisingly, a better understanding of the link between DM and vision loss. Symptoms form triggers for action in participants, as observed in other studies [37, 39, 47]. A qualitative study with PwDM in a high-income setting found that fear of blindness was an incentive to attend DR screening [35], but few of the participants in our study knew that DR was asymptomatic and could lead to blindness.

The importance of understanding the patient within the context of their family, and how this influences patients’ decision-making and actions, has also been observed in the uptake of cataract services in Tanzania. This study showed that the perceived need and mobilisation of resources for cataract surgery was dependent on the family and wider social context [48]. Some studies have also examined the role of the family in DRSS uptake, such as marital status [43], requirement of a person to accompany [49] and household finances [35]. Sri Lanka has a “collectivistic” society and family system, where the needs of the family or a group is considered as a priority over individual needs, as seen in other South Asian countries. Though public health services are free, women defer to men prior to access. Patriarchal norms dictate that the father, husband or the eldest male member plays the central role in earning and decision-making [50, 51]. Older people also rely on family members for addressing their health needs, since there are limited social protection mechanisms [52]. The wider social norms interact with family roles and influences an individual’s health care-seeking behaviour.

Women lack power and authority to attend healthcare services. Previous studies have shown that older women are less motivated to seek eye care, unwilling to use limited family income and reluctant to be a burden on others. This combined with a lack of decision-making power forms significant barriers to access healthcare [53–55]. Family issues such as child care and family attitudes have also formed deterrents to uptake of DRSS in both low-income [24, 25, 37, 56] and high-income countries [35, 42]. So whilst women play a primary role in looking after family health, their own health needs are ancillary. Women’s perceptions of their own needs reinforce the men’s authority in the household. We did not detect any differences in this theme between ethnic groups.

Though men have greater power and independence within the household, our study shows that male PwDM also did not attend screening. Work was a priority, and absence from work formed an opportunity cost in an economy where many participants were earning daily wages, reflecting the lower socioeconomic position of the public clinic patients. The economic role of men in this society contributes to both men and women’s ability to attend healthcare services. Again, the asymptomatic nature of the condition may also contribute to low engagement with screening. Work commitments have been observed in other studies [31]. A study from Hipwell et al. set in the UK found that family attitude and work commitments hinder access [31]. Walker et al. also showed prioritisation of work as a barrier to access [57]. Our sample was drawn from public sector institutes, which provides service for poorer communities, and is consistent with studies where socio-economic position are also determinants of healthcare access [16, 19, 58]. The Western Province has highly a dynamic and industrial economy with significant competition for employment making attendance at work more important than attendance at a screening examination.

This study highlighted a number of institutional-level barriers previously shown in other settings. The eye care services lack capacity in this region, and the clinics are overcrowded. Our participants did not attend an organised screening program, and their appointments were interspersed with other clinic commitments. Consequently, PwDM faced many obstacles and developed negative perceptions about the providers in their experience of DRSS. As described above, economic and family factors suggest that patients would intend to spend a minimum time for DRSS. Most of the participants stated that long waiting times without food was a deterrent for screening attendance. This was a significant concern for PwDM on anti-diabetic medications such as insulin injections with a risk of hypoglycaemia. Similar concerns were raised in a study from the UK [59]. Other institutional barriers such as weak appointment systems [43, 60, 61], time constraints in examination [35, 37, 42], inability to cope with large number of PwDM and [38] less space in screening clinics [62] have also been reported in elsewhere. Discomfort following instillation of pupil-dilating eye drops also discourages attendance at DRSS, in the Western Province and elsewhere [59, 63, 64]. These findings imply that a DRSS should consider using more patient-centred and culturally sensitive strategies.

Access to health care has multiple components beyond healthcare utilisation [65]. Studies have advocated for relevant and culturally competent care delivered to a diverse patient community [20, 66]. The services should be expanded in a way of able to provide universal eye care to PwDM with diverse values, beliefs and behaviours, reducing the disparity. The identification of social norms and other barriers to access DRSS by the PwDM in the Western Province of Sri Lanka highlights both challenges and areas for development. The socio-ecological model enabled us to understand the interactive effects of personal and environmental factors that determined patient access to DRSS [27]. Building on this work, we can use these insights to inform interventions designed to improve uptake of DRSS in this region.

### Limitations

We sampled participants from urban areas attending secondary and tertiary care clinics, and these views do not represent those living in rural areas and attending primary care. The Sri Lankan public health system mainly provides for people from a lower socio-economic background. Therefore, more affluent PwDM are not represented in this study. Since this is a cross section of PwDM population, temporal patterns and seasonal factors may not be reflected. We also recruited low numbers of people from Tamil and Moor ethnic groups, and this may have biased the results and over-represented views from the Sinhala ethnic group. Further exploration with different ethnic groups would be useful to gauge their views in greater depth. Our FGDs were conducted in hospital settings and not in the participants’ own home environment, which may have influenced what participants were willing to say. We selected people attending clinics, and we did not include PwDM who failed to access services completely. However, our sample did capture those who had delayed seeking DRSS and treatment.

## Conclusion

Understanding how DR is conceptualised in this region and responded by the PwDM is essential to define strategies to improve uptake of DRSS. This study shows that there are modifiable barriers to DRSS access in the Western Province of Sri Lanka. These are inter-connected personal, inter-personal, institutional, organisational and environmental barriers which hinder the uptake of DRSS. Availability of DRSS at a convenient location using methods acceptable, culturally and gender sensitive and relevant to PwDM together with strategies to improve the knowledge and awareness among the PwDM may facilitate uptake of screening services in this province.

### Recommendations

Implementation of strategies to improve service availability through a health system approach may be helpful to expand DRSS in this province. There is an urgent need to expand the DRSS in this province with focus on improving waiting times, lengthening consultation periods and developing an organised referral pathway. To address workforce issues, task-shifting or sharing may improve capacity limitations and allow more time for counselling in the busy hospital and clinic settings and reduce waiting times. Our findings indicate health promotion strategies should be focused on engaging with the families of PwDM and their nested environment, in addition to efforts targeted at individual level. Health educational interventions should be gender sensitive and in local languages. A work-based mobile screening approach, i.e. using telemedicine or mobile health (m-health), possibly for larger employers in this region and outreach screening may also improve the coverage of DRSS.

## Additional file

Additional file 1: Topic guide of the focus group discussions. (DOCX 17 kb)

## Abbreviations

DM: Diabetes mellitus
DR: Diabetic retinopathy
DRSS: Diabetic retinopathy screening services
F: Female
FGDs: Focus group discussions
M: Male
M: Moor
PwDM: People with diabetes mellitus
S: Sinhala
T: Tamil
