# Supplementary material for: A qualitative study on barriers and enablers to uptake of diabetic retinopathy screening by people with diabetes in the Western Province of Sri Lanka
Source: Trop Med Health. 2019 May 17;47:34. doi: 10.1186/s41182-019-0160-y (PMC6525343; doi:10.1186/s41182-019-0160-y)
Supplement: Supplementary file 1 — Topic guide of the focus group discussions. (DOCX 17 kb) [file 41182_2019_160_MOESM1_ESM.docx]

**Additional File 1**

**Topic guide of the focus group discussions**

*Topic Guide Version 1.2_Oct_2016*

1. Topic Guide for Conducting the Focus Group Discussions (Service Users)-

Date and Time of the FGD –

Names of the investigators –

Place of the discussion –

Details of the group – (participants’ characteristics data were collected using a questionnaire schedule)

Introduction –

Background information –

*“Burden of the diabetic retinopathy in the Western province of Sri Lanka ……about one fourth (26%) of the total population live with in the Western province of Sri Lanka (5.8 million). The prevalence or the percentage of people having diabetes is high as 18.6% in this province. Available literature / publications show about one third (33%) of the people wit diabetes have any form of retinopathy and about 4% of them are blind due to the same. Diabetes is an emerging public health problem / epidemic in Sri Lanka and it has a significant impact on the health system.*

*A situation analysis done in this province in the year 2014 showed that there was no systematic diabetic retinopathy screening program. It is only an opportunistic screening method. Most of the developed countries have well established screening programs in diabetic retinopathy in order to identify the people who need treatment early. In the Western province, there is a huge gap in the service delivery. Clinicians have experienced that many people present with blindness due to diabetic retinopathy leading to costly surgeries such as pars plana viterctomy and long waiting time for surgeries in the public sector. Therefore it would be a public health concern to identify the reasons for not taking up available services and development of a screening program in the Western province of Sri Lanka. This discussion would be based on this background information and you are allowed to express your views regarding development of a screening program for your province”.*

1) General health care (accessibility and behaviour)

Topic - Where do you usually go for seeking medical care when you fall ill?

Probe - When do you decide to seek medical advice?

2) Specific disease condition (current knowledge, attitude and practice of diabetes)

Topic - Tell me about your condition (diabetes)?

Topic - How did you get to know that you have this condition?

Topic - How do you get treatment for your condition (diabetes)?

3) Perceptions regarding the service providers (medical care)

Topic - What is your opinion on receiving the services and treatment from your health care provider for diabetes?

Prompts - How do you rate the staff members providing the services?

4) Current knowledge regarding the complications of diabetes and medium of receiving information

Topic - What do you know about conditions (complications) that may be associated with long standing diabetes?

Probe - How do you acquire any information or knowledge about those conditions?

5) Patients view about current health educational interventions

Topic - Tell me about the things you have seen, heard or read about the complications of diabetes? Probe - How would you like to acquire this type of information (? through a poster displays at clinics / leaflets/ through newspaper / radio / television / video / from your doctor)

6) Diabetic retinopathy blindness and visual impairment (Current knowledge) –

Topic - Tell me about the things that you know about your condition and associations with your eyes / sight?

7) Diabetic retinopathy - Current attitude and practice

Topic - Tell me about the things that you do about the diabetic eye conditions?

Probe - Have you seen an eye doctor / optician regarding this last few years?

8) Behaviour regarding diabetic retinopathy screening)

Topic - Tell me about the things which may have prevented you from seeing an eye doctor with regard to undergoing an eye examination due to diabetes?

Probe - Was it due to you were not told? You did not have time? Did not have money to go?

Did not like the hospital staff?

(Following questions are specifically for group 3 and 4)

9) Diabetic retinopathy Screening - perceptions regarding the modality of screening -

Topic - What is your opinion about need for checking your eyes as you have diabetes?

Probe - Can you tell me how would you like to check your eyes (method of screening)?

If you have done so, how was the experience at that eye care facility?

10) Follow up

Topic - What is your opinion about check your eyes (for diabetic eye problems) if a doctor suggests it to do regularly?

Topic - How frequent would you like to visit your eye doctor?

11) Barriers in accessing DR treatment services

Topic - What is your opinion about the treatment that you are getting for your eyes for diabetic eye condition?

Topic - Tell me about your experience in undergoing diabetic laser treatment / injections / eye surgery?

(If they have not attended for treatment when required)

Topic - In your opinion what is the main reason for you not undergoing diabetic eye treatments?

(Probes if necessary - Assumptions - was it because you were not aware / expenses / travelling / waiting time / communication problems with the health staff / was it due to you did not like the method of treatment / was it due to the thought that it is not useful since you do not have any eye complains? )

12) Additional

Can you tell me anything that you would like to add to this discussion or any questions that you have regarding checking your eyes (or treating) due to diabetes?
